# Oral hygiene practices and oral health outcomes among older adults in China

**DOI:** 10.3389/fpubh.2026.1748436

**Published:** 2026-02-09

**Authors:** Cheng Han

**Affiliations:** Nanjing Stomatological Hospital, Affiliated Hospital of Medical School, Institute of Stomatology, Nanjing University, Nanjing, China

**Keywords:** brushing frequency, dental caries, older and older adults population, periodontitis, tooth loss

## Abstract

**Background:**

Oral health in older adults is crucial to overall well-being. The high prevalence of dental caries, periodontitis, and tooth loss in older adults Chinese people, together with limited preventative care, highlights the need to investigate oral hygiene behaviors and their effects on oral health.

**Objective:**

To retrospectively evaluate oral hygiene behaviors and their associations with oral health outcomes among older and elder adults in China.

**Methods:**

A retrospective observational study was undertaken on 500 patients aged ≥60 years utilizing hospital data. Demographic characteristics, lifestyle factors, comorbidities, dental hygiene, and oral health outcomes were collected. After adjusting for covariates, chi-square tests and multivariate logistic regression were used to examine oral hygiene behaviors and oral health outcomes.

**Results:**

The average age of participants was 72.4 ± 7.6 years, with 56% female. Most individuals brushed twice daily (66%), used fluoride toothpaste (80%), and had infrequent dental appointments (56% had not seen a dentist in a year). Dental caries, periodontitis, and tooth loss affected 44, 36, and 40% of subjects, respectively. Multivariate analysis showed that twice-daily toothbrushing and the use of fluoride toothpaste were associated with a significant reduction in the risk of dental caries and periodontitis. Bass or circular brushing was associated with a reduced risk of periodontitis (adjusted OR = 0.63, 95% CI: 0.41–0.97). Dental floss, regular dental checkups, and daily mouthwash were associated with reduced tooth loss, gingival bleeding, and halitosis.

**Conclusion:**

Chinese seniors who practiced regular toothbrushing, flossing, and dental visits had better oral health, highlighting the need for improved oral health education and greater emphasis on flossing to support overall oral health in this population.

## 1 Introduction

Dental health is crucial to overall health and quality of life, particularly among older adults (1). Oral health is a critical public health issue as the global geriatric population grows. Poor oral health in older adults is associated to cardiovascular disease, diabetes, malnutrition, respiratory infections, dental caries, periodontitis, tooth loss, and oral mucosal problems (2, 3). These issues can lower the quality of life, social participation, and functioning. Older folks globally have dental difficulties. According to the WHO, almost half of seniors have periodontal disease, and 60–90% suffer from tooth decay. 30–50% of seniors have lost most or all of their teeth, making chewing difficult (4). Chinese older people’s untreated caries, periodontitis, and denture difficulties reflect the country’s growing geriatric population and oral healthcare inadequacies (5). Recent Chinese data suggest that senior persons have terrible oral disease loads that need research. In the 4th National Oral Health Survey, 98.0% of Chinese elders aged 65–74 had untreated dental caries, with at least one tooth rotting, lost, or filled (DMFT ≥ 1) (6, 7). National statistics show 4.5% of Chinese aged people are edentulous and have not received dental prosthetic rehabilitation (8). Just 2.2% of 60-year-olds received professional dental cleanings last year. Geriatric oral hygiene is important for public health, according to Chinese epidemiology. Untreated caries, periodontal disease, and tooth loss are prevalent; behavioral and clinical reasons and preventative strategies are crucial (9, 10).

Biological variables that contribute to dental plaque production include salivary flow, which is related to aging, oral mucosal thinness, and reduced immune response (11, 12). Infrequent toothbrushing, improper toothbrushing technique, limited use of dental floss or mouthwash, infrequent dental visits, smoking, and poor nutrition increase the risk of dental disease (13). Diabetes, cardiovascular disease, dental cavities, and periodontal disease are linked to xerostomia-causing medicines (14, 15). Economic constraints, limited dental treatment, and low health literacy contribute to poor oral hygiene in older persons. Poor oral health in the older adults affects nutrition, systemic health, and quality of life (16, 17). Chewing difficulties and tooth loss can cause malnutrition, poor meal quality, and unexpected weight loss in older persons. Dental infections and periodontitis cause systemic inflammation, which worsens cardiovascular disease and diabetes control (18, 19). Oral infections, speech and swallowing issues, and xerostomia lower quality of life and social participation. Older persons with poor dental hygiene get more respiratory infections, including aspiration pneumonia (20). Dental checkups, flossing, brushing, and mouthwash prevent cavities and gum disease. Dentures and other prostheses require proper care to improve chewing, nutrition, and quality of life. Systemic disorders and healthy habits promote oral health (21, 22). Oral hygiene routines are crucial to oral health, but little is known about their effects on older Chinese adults. This study will retrospectively assess oral hygiene behaviors and oral health outcomes in older Chinese adults to provide clinical and public health initiatives for this vulnerable group.

## 2 Materials and methods

### 2.1 Ethical considerations

This study was approved by the Ethics Committee of Nanjing Stomatological Hospital. No. NJSH-2023NL-37 used only de-identified patient data and involved no direct contact with participants. It was conducted in accordance with the Declaration of Helsinki to ensure ethical standards and confidentiality.

### 2.2 Study design and population

This retrospective observational study examined older people’ dental hygiene habits and health outcomes. Electronic hospital records and patient files provided demographic, clinical, and oral health data. The study initially identified 560 older adults patients from the records of Nanjing Stomatological Hospital. After applying inclusion and exclusion criteria, a total of 500 participants were included in the final analysis. Age ≥ 60 years, complete medical and dental records available in the hospital database, and community-dwelling or institutionalized older adults patients who had at least one documented dental evaluation in the past year were included in this study, and those who had incomplete or missing medical/dental records. Patients with terminal illness or severe cognitive impairment affecting reliable oral health assessment, and those who had major oral or maxillofacial surgery within the past 6 months, were excluded from the study.

### 2.3 Data collection

Data were retrospectively extracted from hospital electronic records and physical patient files using a structured data collection form. Variables collected included demographic information: age, gender, educational level, marital status, living arrangement, employment/retirement status, and residence. Lifestyle factors: smoking status, alcohol consumption, and physical activity level. Comorbidities: cardiovascular disease, hypertension, diabetes mellitus, stroke, and respiratory diseases (e.g., pneumonia, COPD). Oral hygiene practices: frequency and duration of tooth brushing, brushing technique, use of fluoride toothpaste, mouthwash, dental floss, history of dentures or prosthesis, and frequency of dental visits. The structured data collecting form was based on validated instruments used in older adults to assure content validity, and two trained researchers independently assessed a subset of records for reliability and accuracy. This method ensured collected variables were valid and consistent for analysis.

### 2.4 Data analysis

Data were entered and analyzed using statistical software (SPSS version 25). Descriptive statistics were generated, including mean ± SD for continuous variables and frequencies (%) for categorical variables. Using chi-square tests for categorical variables and logistic regression models to generate ORs with 95% CIs, gender, age, comorbidities, and lifestyle factors were used to analyze oral hygiene habits and oral health outcomes.

## 3 Results and discussion

### 3.1 Demographic and health characteristics of the study population

The study included 500 older adults volunteers with a mean age of 72.4 years (SD = 7.6 years). 16% of the participants were 60–64 years old, 22% were 65–69 years old, 24% were 70–74 years old, 18% were 75–79 years old, 12% were 80–84 years old, and 8% were 85 years old. The population was 44% male and 56% female; 30% of the population had no formal education, 36 % had completed elementary, 24 % had completed secondary, and 10 % had completed higher education; 6% were divorced, 30 % widowed, and 64 % married; 74% lived with family, 20 % alone, and 6 % in institutions. Lifestyle characteristics showed that 56% of individuals had never smoked, 30% had smoked in the past, and 14% smoked currently; 70% never drank, 20 % occasionally, and 10 % often; 40% were idle, 44 % somewhat active, and 16 % exercised. Three-sixths of patients had hypertension, 24% had cardiovascular disease, 16% had diabetes, 8% had strokes, and 10% had respiratory problems such as pneumonia or COPD (Table 1).

**Table 1:** Demographic and health characteristics of study population.

Characteristic	Subcategory/range	*n* (%) or mean ± SD
Total participants		500 (100)
Age (years)	Mean ± SD	72.4 ± 7.6
Age group	60–64	80 (16)
	65–69	110 (22)
	70–74	120 (24)
	75–79	90 (18)
	80–84	60 (12)
	≥85	40 (8)
Gender	Male	220 (44)
	Female	280 (56)
Educational level	No formal education	150 (30)
	Primary school	180 (36)
	Secondary school	120 (24)
	Higher education	50 (10)
Marital status	Married	320 (64)
	Widowed	150 (30)
	Divorced/separated	30 (6)
Living arrangement	Living alone	100 (20)
	Living with spouse/family	370 (74)
	Living in institutional care	30 (6)
Employment/retirement status	Employed	60 (12)
	Retired	410 (82)
	Unemployed	30 (6)
Residence	Urban	300 (60)
	Rural	200 (40)
Lifestyle factors	Smoking – never	280 (56)
	Smoking – former	150 (30)
	Smoking – current	70 (14)
	Alcohol consumption – never	350 (70)
	Alcohol consumption – occasional	100 (20)
	Alcohol consumption – regular	50 (10)
	Physical activity – sedentary	200 (40)
	Physical activity – moderate	220 (44)
	Physical activity – active	80 (16)
Comorbidities	Cardiovascular disease (CVD)	120 (24)
	Hypertension	180 (36)
	Diabetes mellitus	80 (16)
	Stroke	40 (8)
	Respiratory disease (pneumonia, COPD)	50 (10)

*n* (%), number and percentage of participants; SD, standard deviation.

### 3.2 Oral hygiene practices of the study population

Among the 500 older persons, a wide range of oral hygiene practices was observed. Among the people who took part in the research, 66 % rinsed their mouths, 24 % brushed their teeth, and 66 % cleaned their teeth at least three times a day; 64% of the participants cleaned their teeth for 1 to 2 min, 24 % for longer, and 12 % for shorter; 1 to 2 min was the typical amount of time spent brushing one’s teeth. There were 50 % of people who used the vertical/bass brushing approach, 30 % who used the horizontal technique, and 20 % who used the circular technique. Fluoride toothpaste was used by 80 % of the people who participated in the study, whereas 20 % did not use it. It was found that 24% of people used mouthwash on a regular basis, 36% used it occasionally, and 40% never used it. In addition, only 10% of people used dental floss on a regular basis, 24% used it rarely, and 66% never used it. The percentage of respondents who had visited a dentist on a regular basis in the preceding year was 44%, while the percentage of respondents who had not seen a dentist was 56%. Two-thirds of patients came in for routine examinations, while 16% came in for treatment or discomfort related to their dental health. There were 10% of respondents who said that their oral health was exceptional, 40% who said it was good, 36% who said it was fair, and 14% who said it was poor. A total of 30 % of the individuals in the study had previously utilized dentures or prostheses, whereas 70 % had not (Table 2).

**Table 2:** Detailed oral hygiene practices.

Oral hygiene practice (*n* = 500)	Subcategory/Frequency	*n* (%)
Tooth brushing frequency	Once daily	120 (24)
	Twice daily	330 (66)
	≥3 times daily	50 (10)
Tooth brushing duration	<1 min	60 (12)
	1–2 min	320 (64)
	>2 min	120 (24)
Tooth brushing technique	Horizontal	150 (30)
	Vertical/Bass	250 (50)
	Circular	100 (20)
Use of fluoride toothpaste	Yes	400 (80)
	No	100 (20)
Use of mouthwash	Daily	120 (24)
	Occasionally	180 (36)
	Never	200 (40)
Use of dental floss	Daily	50 (10)
	Occasionally	120 (24)
	Never	330 (66)
Regular dental visits (past year)	Yes	220 (44)
	No	280 (56)
Reasons for last dental visit	Routine check-up	140 (28)
	Dental pain/treatment	80 (16)
Self-reported oral health status	Excellent	50 (10)
	Good	200 (40)
	Fair	180 (36)
	Poor	70 (14)
History of dentures/prosthesis	Yes	150 (30)
	No	350 (70)

*n* (%), number and percentage of participants.

## 4 Oral health outcomes among participants

A study of 500 older adults found that dental diseases were common. The older participants had many dental problems; 44% had dental caries, 36 % had periodontitis, and 40 % had lost teeth; 30% had gingiva hemorrhages, and 6 % had ulcers or leukoplakia. Halitosis was noted by 20% and dry mouth by 18%. Also prominent were oral health-related functioning deficits. Oral issues caused 8% trouble swallowing, 26% difficulty chewing, and 7% difficulty speaking. These were dental issues. Mouth pain reduced meal intake for 20% of respondents. Dentures were worn by 24, 16, and 8% of people. Of participants, 24% wore dentures. This study explains tooth loss and orthodontic therapy in this demographic (Table 3).

**Table 3:** Oral health outcomes among participants.

Oral health outcome category (*n* = 500)	Specific outcome/Description	*n* (%)
Dental conditions	Dental caries	220 (44)
	Periodontitis	180 (36)
	Tooth loss (number of teeth missing ≥1)	200 (40)
	Gingival bleeding	150 (30)
	Oral mucosal lesions (ulcers, leukoplakia)	30 (6)
	Halitosis (self-reported)	100 (20)
	Dry mouth/xerostomia	90 (18)
Functional outcomes	Difficulty chewing/masticatory problems	130 (26)
	Difficulty swallowing (dysphagia)	40 (8)
	Speech difficulties due to dental issues	35 (7)
	Reduced dietary variety due to oral discomfort	100 (20)
Prosthetic status	Denture use	120 (24)
	Partial dentures	80 (16)
	Full dentures	40 (8)

*n* (%), number and percentage of participants.

### 4.1 Associations between oral hygiene practices and oral health outcomes

Oral hygiene and oral health were found to be significantly associated, according to the findings of an investigation. It was found that brushing teeth once daily was related to a lower risk of dental caries (odds ratio = 0.48, 95% confidence interval: 0.33–0.70, *p* < 0.001) and periodontitis (odds ratio = 0.52, 95% confidence interval: 0.36–0.76, *p* = 0.001) in comparison to brushing teeth twice daily. There was a significant reduction in tooth loss with the use of dental floss (odds ratio = 0.55, 95% confidence interval: 0.34–0.87, *p* = 0.010). The odds ratio was 0.60, the 95% confidence interval was 0.42–0.87, and the *p*-value was 0.006. Regular dental visits were found to minimize gingival bleeding. Daily use of mouthwash was associated with reduced halitosis (odds ratio = 0.50, 95% confidence interval: 0.30–0.82, *p* = 0.005). Fluoride toothpaste significantly reduced the risk of dental caries (odds ratio = 0.45; 95% confidence interval, 0.28–0.72; *p* < 0.001). Bass or circular brushing reduced periodontitis risk compared to horizontal brushing (odds ratio = 0.65; 95% CI, 0.42–1.00; *p* = 0.05). According to these research, older individuals’ oral health is closely associated to brushing, flossing, fluoride usage, and regular dental care (Table 4).

**Table 4:** Association between oral hygiene practices and oral health outcomes.

Oral hygiene practice	Oral health outcome	OR (95% CI)	*p*-value
Brushing twice daily vs. once	Dental caries	0.48 (0.33–0.70)	<0.001
Brushing twice daily vs. once	Periodontitis	0.52 (0.36–0.76)	0.001
Use of dental floss vs. none	Tooth loss	0.55 (0.34–0.87)	0.01
Regular dental visits vs. none	Gingival bleeding	0.60 (0.42–0.87)	0.006
Mouthwash use (daily) vs. never	Halitosis	0.50 (0.30–0.82)	0.005
Fluoride toothpaste vs. non-fluoride	Dental caries	0.45 (0.28–0.72)	<0.001
Brushing technique (Bass/circular) vs. horizontal	Periodontitis	0.65 (0.42–1.00)	0.05

OR, odds ratio; CI, confidence interval; reference groups – brushing once daily, no dental floss use, no regular dental visits, never using mouthwash, non-fluoride toothpaste, and horizontal brushing technique. Statistically significant associations are indicated by *p*-values < 0.05.

### 4.2 Multivariate regression analysis for oral health outcomes

A multivariate logistic regression was performed to identify oral health factors among older adults. Double-daily brushing was linked to lower risk of dental caries (*β* = −0.69, SE = 0.18; adjusted odds ratio = 0.50, 95% confidence interval: 0.34–0.73, *p* < 0.001) and periodontitis (*β* = −0.60, SE = 0.20; adjusted odds ratio = 0.55, 95% confidence interval: 0.38–0.80, *p* = 0.002). Fluoride toothpaste was found to significantly protect against dental caries (*β* = −0.76, standard error = 0.24; adjusted odds ratio = 0.47, 95% confidence interval: 0.29–0.75, *p* = 0.002). A strong correlation exists between brushing techniques, particularly the Bass or circular method, and reduced incidence of periodontitis (*β* = −0.46, SE = 0.22; adjusted odds ratio = 0.63, 95% confidence interval: 0.41–0.97, *p* = 0.04). Dental floss use reduced tooth loss risk (*β* = −0.55, SE = 0.23; adjusted odds ratio = 0.58, 95% confidence interval: 0.36–0.91, *p* = 0.02), while regular dental visits reduced gingival bleeding risk (*β* = −0.49, SE = 0.18; adjusted odds ratio = 0.61, 95% confidence interval: 0.42–0.88, *p* = 0.007). The study found that daily mouthwash use reduced the probability of halitosis (*β* = −0.67, SE = 0.23; adjusted odds ratio = 0.51, 95% confidence interval: 0.31–0.84, *p* = 0.007) (Table 5). This discovery is significant. From these findings, older persons can dramatically lower their risk of common oral health issues by practicing good oral hygiene and getting preventative dental care.

**Table 5:** Multivariate regression analysis for oral health outcomes.

Oral Health Outcome	Predictor	β (SE)	Adjusted OR (95% CI)	*p*-value
Dental caries	Brushing twice daily vs. once	−0.69 (0.18)	0.50 (0.34–0.73)	<0.001
	Fluoride toothpaste use	−0.76 (0.24)	0.47 (0.29–0.75)	0.002
Periodontitis	Brushing technique (Bass/circular)	−0.46 (0.22)	0.63 (0.41–0.97)	0.04
	Brushing twice daily vs. once	−0.60 (0.20)	0.55 (0.38–0.80)	0.002
Tooth loss	Use of dental floss vs. none	−0.55 (0.23)	0.58 (0.36–0.91)	0.02
Gingival bleeding	Regular dental visits vs. none	−0.49 (0.18)	0.61 (0.42–0.88)	0.007
Halitosis	Mouthwash daily vs. never	−0.67 (0.23)	0.51 (0.31–0.84)	0.007

*β*, regression coefficient; SE, standard error; OR, odds ratio; CI, confidence interval. Reference groups, brushing once daily, nonfluoride toothpaste, horizontal brushing technique, no flossing, no regular dental visits, and never using mouthwash. Statistically significant associations are indicated by *p*-values < 0.05.

## 5 Discussion

This study aligns with Chinese research on the older adults. Longer life expectancy among women has led to a higher proportion of women in similar research. Previous generations of Chinese older adults have had limited access to formal education, which explains their low educational attainment. This study found marital status and living arrangements similar to those reported in previous community-based surveys, suggesting that co-residence within the family may provide social and health support (23). Smoking, alcohol use, and physical activity are consistent with national and regional studies of Chinese older populations, which show high sedentary behavior and low habitual exercise. Epidemiological surveys show that older persons have a significant burden of chronic non-communicable disorders such as hypertension, cardiovascular disease, and diabetes. Rural Chinese elder research reported a significant incidence of cardiometabolic disorders like hypertension (74.8%) and diabetes (14.9%), especially among women (24). Another Longitudinal Healthy Longevity Survey study found that more than two-thirds of older adults had multimorbidity, and that education was a major risk factor for impairment (25). Research shows that oral health examinations should be part of overall health management in this population.

Research in China and elsewhere indicated that seniors’ dental hygiene regimens were comparable to those of younger generations. Investigators examined participants. Although toothbrushing twice daily is the most common practice, many older Chinese adults brush their teeth less frequently or inadequately. A poll indicated that 80% of people use fluoride toothpaste. Because this statistic is slightly higher than that reported in the regional study, urban and semi-urban residents may be more aware of the benefits of fluoride toothpaste. Previous research has shown that older adults in China rarely use dental floss due to reduced manual dexterity, cost, and limited understanding of its benefits. These findings support 10% of persons flossing daily (26). Professional dental visit rates (44%) are comparable to those reported in earlier studies, indicating that older Chinese adults seek dental care only when ill. Self-reported oral health status and denture use patterns also support epidemiological studies indicating a high incidence of untreated oral disease and the need for improved preventive care in this population (27). In a Mexican cross-sectional study of older adults (≥60), only 3.6% used dental floss and 16.5% used mouthwash, indicating low utilization of interdental cleaning aids (28). According to a study of older community residents, 42 percent washed their teeth twice daily, whereas only 36 percent flossed or brushed their teeth frequently (29). Older people typically brush their teeth, but global surveys show minimal flossing and mouthwash use. This suggests the need for greater oral health education and treatment.

This work supports research on ageing populations in China and globally. National surveys indicate that 44% of older adults have dental caries and 36% have periodontitis. Previous Chinese research has found that many older adults persons have at least one missing tooth, typically without prosthetic replacement. 40% of participants lost teeth (30). Regional studies show age-related salivary changes, systemic illnesses, and poor oral hygiene cause comparable rates of gingival bleeding (30%) and xerostomia (18%). Chewing (26%) and dietary diversity (20%) limitations among older persons, due to tooth loss and untreated oral illness, affect nutrition and quality of life (13, 31). Other research indicates that cost and access barriers limit prosthesis rehabilitation for many older adults with tooth loss. Denture use (24% of the population) and the distribution of partial and complete dentures confirm these findings (32). Numerous nations have made similar observations. According to the Swedish National Study on Ageing and Care, the prevalence of periodontitis increases with age. After investigating, researchers found. Despite regular dental appointments, nearly half of the “old-old” group aged 81 lost teeth and bone (33). According to US studies, 68% of 65-year-olds had chronic periodontitis (34). Global studies indicate that oral disease among older persons is widespread, including in China, underscoring the need for preventive measures, routine dental care, and effective prosthesis rehabilitation.

These relationships have been confirmed by Chinese and international aging studies. A new study shows that twice-daily fluoride toothpaste brushing decreases periodontal disease and caries. Our data support Tables 4, 5 showing twice-daily brushing lowers dental caries and periodontitis due to better plaque removal and bacterial reduction than once-daily brushing. Regular dental checkups and flossing can detect and eliminate plaque early, preventing gingival irritation and tooth loss. Interproximal plaque reduction may explain why daily dental flossing reduced tooth loss. Due to its remineralization and acid-producing bacteria suppression, fluoride toothpaste protected against dental cavities. Bass (circular) brushing may have lowered periodontitis risk via improved gingival edge cleansing. Regular dental visits reduced gingival bleeding, possibly due to inflammation identification and treatment. Halitosis may be minimized by daily mouthwash use, which lowers oral bacterial burden and volatile sulfur compounds (21, 35). A study of US adults over 65 found that flossers had decreased incidences of periodontal disease, cavities, and tooth loss over 5 years (36). In a randomized clinical trial, brushing and rinsing with mouthwash that contains cetylpyridinium chloride (CPC) may help to reduce oral malodor (halitosis) and volatile sulfur compound-producing bacteria better than brushing alone (37).

### 5.1 Limitation of the study

This study is limited by its retrospective design, potential selection bias toward urban and semi-urban older adults, exclusion of the rural group, and a binary oral health outcome analysis that does not account for clinical severity or longitudinal change. Nutrition, socioeconomic status, and access to non-hospital dental care may have affected the reported associations.

## 6 Conclusion

This study demonstrates that oral hygiene behavior and preventive care substantially affect the oral health status of older Chinese adults, among whom dental caries, periodontitis, tooth loss, and functional impairments remain highly prevalent. In addition to routine dental examination and twice-daily toothbrushing with fluoride toothpaste, the findings underscore the critical role of oral health education in promoting self-care practices. Promotion of appropriate brushing techniques (e.g., circular or Bass methods), interdental cleaning, and the use of therapeutic mouthwashes can improve oral microbial control, halitosis, masticatory function, nutritional status, and overall quality of life. Public health strategies should therefore prioritize culturally appropriate oral health education, accessible preventive services, and targeted behavioral and clinical interventions to reduce the burden of oral disease and improve the well-being of older adults.

## Data Availability

The original contributions presented in the study are included in the article/Supplementary material, further inquiries can be directed to the corresponding author.
