# Supplementary material for: Oral hygiene practices and oral health outcomes among older adults in China
Source: Front Public Health. 2026 Feb 9;14:1748436. doi: 10.3389/fpubh.2026.1748436 (PMC12926170; doi:10.3389/fpubh.2026.1748436)
Supplement: Supplementary file 1 [file Table_1.docx]

Table: Multivariate Regression Analysis of Oral Health Outcomes – Including Non-Significant Findings

| **Oral Health Outcome** | **Predictor** | **β (SE)** | **Adjusted OR (95% CI)** | **p-value** | **Significance** |
| --- | --- | --- | --- | --- | --- |
| Dental caries | Brushing twice daily vs once | -0.69 (0.18) | 0.50 (0.34–0.73) | <0.001 | Yes |
| Dental caries | Fluoride toothpaste use | -0.76 (0.24) | 0.47 (0.29–0.75) | 0.002 | Yes |
| Dental caries | Use of dental floss vs none | -0.10 (0.20) | 0.90 (0.60–1.34) | 0.65 | No |
| Dental caries | Mouthwash use daily vs never | -0.08 (0.19) | 0.92 (0.63–1.34) | 0.70 | No |
| Dental caries | Regular dental visits vs none | -0.12 (0.18) | 0.89 (0.63–1.26) | 0.52 | No |
| Dental caries | Brushing technique (Bass/circular vs horizontal) | -0.20 (0.22) | 0.82 (0.54–1.26) | 0.38 | No |
| Periodontitis | Brushing twice daily vs once | -0.60 (0.20) | 0.55 (0.38–0.80) | 0.002 | Yes |
| Periodontitis | Brushing technique (Bass/circular vs horizontal) | -0.46 (0.22) | 0.63 (0.41–0.97) | 0.04 | Yes |
| Periodontitis | Fluoride toothpaste use | -0.10 (0.22) | 0.90 (0.60–1.34) | 0.65 | No |
| Periodontitis | Dental floss use vs none | -0.08 (0.21) | 0.92 (0.63–1.34) | 0.70 | No |
| Periodontitis | Mouthwash daily vs never | -0.06 (0.20) | 0.94 (0.64–1.38) | 0.75 | No |
| Periodontitis | Regular dental visits | -0.15 (0.19) | 0.86 (0.60–1.23) | 0.42 | No |
| Tooth loss | Use of dental floss vs none | -0.55 (0.23) | 0.58 (0.36–0.91) | 0.02 | Yes |
| Tooth loss | Brushing twice daily | -0.18 (0.19) | 0.84 (0.59–1.18) | 0.33 | No |
| Tooth loss | Fluoride toothpaste | -0.12 (0.21) | 0.89 (0.61–1.31) | 0.55 | No |
| Tooth loss | Mouthwash daily | -0.10 (0.20) | 0.90 (0.63–1.29) | 0.65 | No |
| Tooth loss | Regular dental visits | -0.09 (0.18) | 0.91 (0.64–1.28) | 0.59 | No |
| Gingival bleeding | Regular dental visits | -0.49 (0.18) | 0.61 (0.42–0.88) | 0.007 | Yes |
| Gingival bleeding | Brushing twice daily | -0.10 (0.20) | 0.90 (0.62–1.32) | 0.65 | No |
| Gingival bleeding | Fluoride toothpaste | -0.08 (0.22) | 0.92 (0.61–1.38) | 0.70 | No |
| Gingival bleeding | Dental floss use | -0.12 (0.21) | 0.89 (0.60–1.33) | 0.65 | No |
| Gingival bleeding | Mouthwash daily | -0.06 (0.19) | 0.94 (0.65–1.36) | 0.72 | No |
| Halitosis | Mouthwash daily vs never | -0.67 (0.23) | 0.51 (0.31–0.84) | 0.007 | Yes |
| Halitosis | Brushing twice daily | -0.12 (0.19) | 0.89 (0.63–1.27) | 0.55 | No |
| Halitosis | Fluoride toothpaste | -0.10 (0.20) | 0.90 (0.63–1.29) | 0.65 | No |
| Halitosis | Dental floss use | -0.08 (0.21) | 0.92 (0.64–1.33) | 0.70 | No |
| Halitosis | Regular dental visits | -0.11 (0.19) | 0.90 (0.64–1.27) | 0.58 | No |
